# *FBXO7* Y52C Polymorphism as a Potential Protective Factor in Parkinson's Disease

**DOI:** 10.1371/journal.pone.0101392

**Published:** 2014-07-16

**Authors:** Chiung-Mei Chen, I-Cheng Chen, Yi-Cheng Huang, Hsueh-Fen Juan, Ying-Lin Chen, Yi-Chun Chen, Chih-Hsin Lin, Li-Ching Lee, Chi-Mei Lee, Guey-Jen Lee-Chen, Yun-Ju Lai, Yih-Ru Wu

**Affiliations:** 1 Department of Neurology, Chang-Gung Memorial Hospital, Chang-Gung University College of Medicine, Taipei, Taiwan; 2 Department of Life Science, National Taiwan Normal University, Taipei, Taiwan; 3 Department of Life Science, National Taiwan University, Taipei, Taiwan; Centre Hospitalier Universitaire Vaudois (CHUV), Switzerland

## Abstract

Mutations in the F-box only protein 7 gene (*FBXO7*), the substrate-specifying subunit of SCF E3 ubiquitin ligase complex, cause Parkinson's disease (PD)-15 (PARK15). To identify new variants, we sequenced *FBXO7* cDNA in 80 Taiwanese early onset PD patients (age at onset ≤50) and only two known variants, Y52C (c.155A>G) and M115I (c.345G>A), were found. To assess the association of Y52C and M115I with the risk of PD, we conducted a case–control study in a cohort of PD and ethnically matched controls. There was a nominal difference in the Y52C G allele frequency between PD and controls (*p* = 0.045). After combining data from China [1], significant difference in the Y52C G allele frequency between PD and controls (*p* = 0.012) and significant association of G allele with decreased PD risk (*p* = 0.017) can be demonstrated. Upon expressing EGFP-tagged Cys52 FBXO7 in cells, a significantly reduced rate of FBXO7 protein decay was observed when compared with cells expressing Tyr52 FBXO7. *In silico* modeling of Cys52 exhibited a more stable feature than Tyr52. In cells expressing Cys52 FBXO7, the level of TNF receptor-associated factor 2 (TRAF2) was significantly reduced. Moreover, Cys52 FBXO7 showed stronger interaction with TRAF2 and promoted TRAF2 ubiquitination, which may be responsible for the reduced TRAF2 expression in Cys52 cells. After induced differentiation, SH-SY5Y cells expressing Cys52 FBXO7 displayed increased neuronal outgrowth. We therefore hypothesize that Cys52 variant of FBXO7 may contribute to reduced PD susceptibility in Chinese.

## Introduction

Parkinson's disease (PD) is attributable to environmental factors, whereas evidence has shown that genetic factors are also playing an important role. Molecular genetic studies have identified thirteen genes linked to rare dominant or recessive monogenic forms of PD: *SNCA*, *Parkin*, *PINK1*, *DJ-1*, *LRRK2*, *ATP13A2*, *VPS35*, *PLA2G6*, *FBXO7*, *EIF4G1*, *SYNJ1*, *DNAJC6*, and *DNAJC13* [2], [3].

Recently, the F-box only protein 7 (*FBXO7*) mutations have been identified in several families with early-onset parkinsonism and pyramidal tract signs. Homozygous R378G missense mutation in an Iranian kindred, homozygous nonsense mutation (R498X) in an Italian family, a Pakistan family and a Turkey family, and compound heterozygous mutations (IVS7+1G/T and T22M) in a Dutch family are unambiguously responsible for the autosomal recessive, early-onset, parkinsonian-pyramidal syndrome [4], [5], [6]. The presynaptic nature of the parkinsonism in the families reported by Di Fonzo et al. is shown by the dramatic abnormality of DaTSCAN SPECT, the beneficial effect of levodopa, and the presence of levodopa-induced dyskinesias, suggesting that *FBXO7* mutations may be the potential genetic causes of early-onset Parkinson's disease (EOPD) or familial PD [5]. Two missense substitutions, p.Ile87Thr and p.Asp328Arg, in a single heterozygous state, were found in two EOPD patients in Taiwan [7]. Although no pathogenetic mutations in the *FBXO7* gene were detected in 135 Chinese early-onset parkinsonism patients, the PD patients showed a trend toward decrease in Y52C G allele frequency compared with the controls [1].

*FBXO7*, a member of the F-box-containing protein (FBP) family, encodes a protein of 522 amino acids consisting of several discrete domains: the ubiquitin-like domain, cyclin-dependent protein kinase 6 (CDK6) binding site, FBXO7/PI31 domain, F-box motif, proline-rich region, and R(ar)DP motif [5], [8]. Through the interaction between the F-box and the Skp1 protein, FBPs become part of SCF (Skp1-Cullin1-F-box protein) ubiquitin ligase complexes, and play roles in ubiquitin-mediated proteasomal degradation (review in [9]). F-box proteins recruit a large number of diverse substrates to SCF complexes and allow for their ubiquitination [10]. FBXO7 promotes ubiquitin conjugation to TRAF2 (a member of the tumor necrosis factor receptor associated factor protein family with ubiquitin ligase activity) and cIAP1 (an apoptosis inhibitor possessing ubiquitin ligase activity), resulting in decreased receptor-interacting protein 1 (RIP1) ubiquitination and lowered NF-κB signaling activity [11], [12]. Strong evidence has shown that NF-κB induced neuroinflammation may be involved in development of PD [13], [14]. Therefore, FBXO7 may play a role in protecting neurons from PD process. In contrast, the mutations or variations that change the function, expression, or stability of FBXO7 may confer PD risk to the subjects.

## Results

### Mutation/variant analysis of *FBXO7*

**Figure 1:**
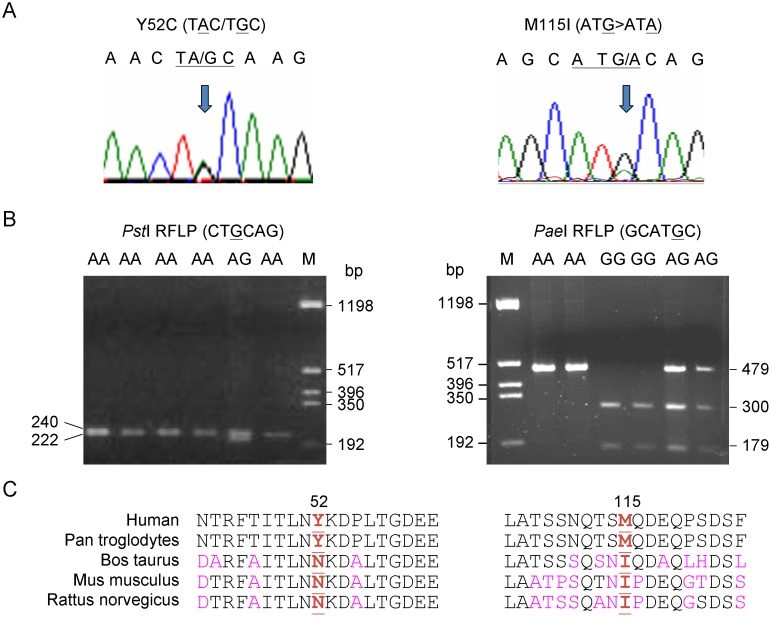
Variant identification and amino acid sequence alignment. (A) Chromatograms of direct cDNA sequencing of Y52C and M115I. (B) Restriction analysis of Y52C and M115I. On agarose gel, Y52C creates new *Pst*I restriction site by mismatch PCR and leads to an additional 222 bp band, whereas M115I loses restriction by *Pae*I and leads to 479 bp band. (C) Evolutionary conservation of the regions of FBXO7 Y52C and M115I using the program Vector NTI.

Since most of the mutations found in *FBXO7* result in truncated FBXO7 protein, amino acid replacement or multiple aberrant frame-shift splice variants, we sequenced *FBXO7* cDNA (Table 1) instead of genomic DNA in a cohort of ethnic Chinese patients with EOPD in Taiwan to identify previously undetected variants in EOPD cases of Chinese origin, followed by a case–control study for the identified variants. The cDNA samples contained only DNA sequences from genes that were transcribed into RNA. Thus *FBXO7* cDNA fragments from 80 EOPD patients were amplified for sequence analysis. However, only two known substitutions that caused changes in the peptide sequence were identified: a c.155A>G substitution leading to an amino acid change from tyrosine to cysteine in position 52 (Y52C) in one EOPD patient (heterozygote) and a c.345G>A substitution resulting in a methionine to isoleucine change at amino acid position 115 (M115I) (rs11107) in 74 EOPD patients (40 homozygotes and 34 heterozygotes) (amino acid number according to NM_012179) (Fig. 1A). The two reported [1] variants were confirmed using PCR-restriction fragment length polymorphism (RFLP) method (Fig. 1B). Both Y52C and M115I are not evolutionarily conserved in the known mammalian homologues of the FBXO7 protein (Fig. 1C).

**Table 1:** Primers and conditions for PCR amplification of *FBXO7* cDNA and genomic DNA.

		Product/
	Anneal (°C)/	RFLP enzyme
Test (amplified region)	MgCl_2_ (mM)	(fragment, bp)
cDNA sequencing		
F: CTCTTTCCCCGTTTCGCC	58/1.5	1955
R: GGAGAACCAAGAGCAGGGAGA		
pEGFP-N1-FBXO7 cDNA cloning		
F: AAGCTT CTCTTTCCCCGTTTCGCCTCAG	62/1.5	1727
R: ACCGGTGGCATGAATGACAGCCGGCC		
pcDNA3.1/V5-His-FBXO7 cloning		
F: AAGCTT CTCTTTCCCCGTTTCGCCTCAG	62/1.5	1726
R: CTCGAG ACATGCATGACAGCCGGCCATC		
Y52C (TAC/TGC)		
F: AGGCTGAGGCAGGAGGAT TG	58/1.5	*Pst*I: CTGCAG
R: CTCCAGTGAGGGGATCCCTG		(240/222, 18)
M115I (ATG/ATA)		
F: TCACTGGAGATGAAGAGACC	58/1.5	*Pae*I: GCATGC
R: AATCGCTTGAACTGGGAGGC		(300, 179/479)

The *Pst*I restriction site was created by PCR using a mismatch primer. For Y52C and M115I amplification, the underlines in the primer sequence and enzyme recognition site indicate the mismatch nucleotide and polymorphic site, respectively. For cDNA cloning, the underlines in the primer sequence indicate the introduced *Hin*dIII, *Age*I and *Xho*I restriction sites.

### Case–control study of Y52C and M115I

A case–control study in a cohort of PD patients (n = 516, 80 EOPD patients included) and ethnically matched controls (n = 516) was conducted to assess the association of Y52C and M115I with the risk of PD (Table 2). All genotype frequencies were confirmed to be in the Hardy–Weinberg equilibrium. There was no statistically significant difference (*p*>0.025) in genotype or allele distribution between patients and controls for both single nucleotide polymorphisms (SNPs) examined, after correction of multiple SNP testing. However, for Y52C, the frequency of AG genotype (0.8% vs. 2.3%, *p* = 0.044) or G allele (0.4% vs. 1.2%, *p*  = 0.045) was notably lower in PD patients than the controls. Y52C AG genotype or G allele demonstrated a trend toward decrease in risk of developing PD (odds ratio: 0.33, 95% confidence interval: 0.09–0.95, *p* = 0.055–0.056). Analysis combining our patient and control subjects as well as the population in Luo's study [1] yielded results of statistically significant difference in genotype (0.9% vs. 2.8%, *p* = 0.012) and allele (0.5% vs. 1.4%, *p* = 0.012) distribution between patients and controls. The negative association of the Y52C AG genotype or G allele with PD was significant (odds ratio: 0.32–0.33, 95% confidence interval: 0.12–0.77, *p* = 0.016–0.017). The identified one EOPD and three late-onset PD patients carrying Y52C presented with asymmetrical tremor, rigidity, and bradykinesia without pyramidal signs, all of whom had a good response to anti-parkinsonian medication.

**Table 2:** Genotype and allele distributions and association analysis.

		No. (%)		Odds ratio (95% CI)	*p*-Value
		PD	Control		
Genotype/allele					
Y52C (TAC/TGC)					
	AA	512 (99.2)	504 (97.7)	1.00	
	AG	4 (0.8)	12 (2.3)	0.33 (0.09–0.95)	0.055
	Locus total *p*		0.044		
	A	1028 (99.6)	1020 (98.8)	1.00	
	G	4 (0.4)	12 (1.2)	0.33 (0.09–0.95)	0.056
	Locus total *p*		0.045		
M115I (ATG/ATA)					
	AA	258 (50.0)	258 (50.0)	1.00	
	AG	220 (42.6)	221 (42.8)	1.00 (0.77–1.28)	0.972
	GG	38 (7.4)	37 (7.2)	1.03 (0.63–1.67)	0.914
	Locus total *p*		0.992		
	A	736 (71.3)	737 (71.4)	1.00	
	G	296 (28.7)	295 (28.6)	1.00 (0.83–1.22)	0.961
	Locus total *p*		0.961		
Y52C (TAC/TGC) (Luo et al., 2010)					
	AA	133 (98.5)	192 (96.0)	1.00	
	AG	2 (1.5)	8 (4.0)	0.36 (0.05–1.47)	0.202
	Locus total *p*		0.184		
	A	268 (99.3)	392 (98.0)	1.00	
	G	2 (0.7)	8 (2.0)	0.37 (0.06–1.47)	0.201
	Locus total *p*		0.187		
Y52C Combined [Taiwan + China (Luo et al., 2010)]					
	AA	645 (99.1)	696 (97.2)	1.00	
	AG	6 (0.9)	20 (2.8)	0.32 (0.12–077)	0.016
	Locus total *p*		0.011		
	A	1296 (99.5)	1412 (98.6)	1.00	
	G	6 (0.5)	20 (1.4)	0.33 (0.12–0.77)	0.017
	Locus total *p*		0.012		

Odds ratios were calculated by comparing each value with the major common genotype or allele.

### FBXO7 expression analysis

Since the case–control study suggests that *FBXO7* Y52C G allele might be a potential protective factor, we cloned the polymorphic *FBXO7* cDNA, which was then expressed in HEK-293T cells to investigate the functional consequences.

**Figure 2:**
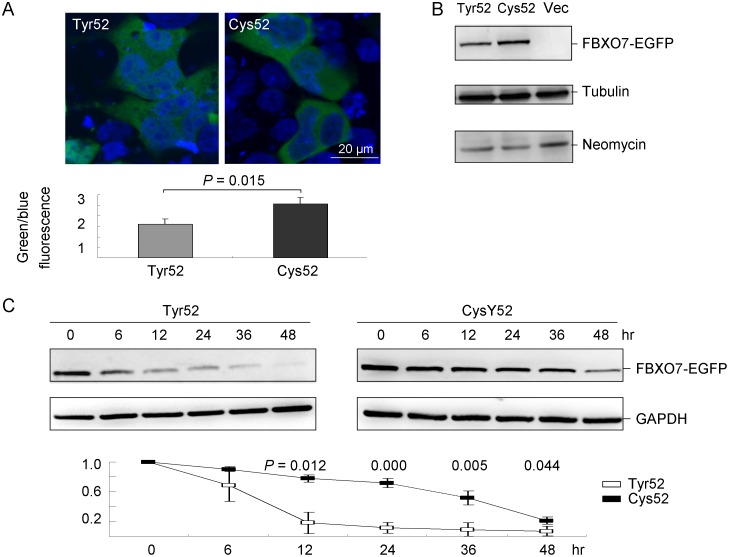
Expression of FBXO7 protein in HEK-293T cells. (A) Confocal microscopy examination of Tyr52 and Cys52 FBXO7-EGFP protein in HEK-293T cells. HEK-293T cells were transfected with Tyr52 and Cys52 FBXO7-EGFP for two days and stained with DAPI to detect nuclei. The green (FBXO7 fusion protein) to blue (nuclei staining) fluorescence signal was quantified and shown below. (B) Western blot analysis of pEGFP-N1 vector (Vec) and Tyr52 (WT) and Cys52 FBXO7-EGFP transfected cells using FBXO7 antibody and anti-tubulin and anti-neomycin antibodies as loading and transfection controls, respectively. (C) Tyr52 and Cys52 FBXO7-EGFP proteins in transfected cells were analyzed after blocking of new protein synthesis in a cycloheximide (200 µg/ml) chase experiment (cells were harvested after 6, 12, 24, 36, and 48 hr). Data are represented as the means ± S.D. of three separate experiments.

The common cellular abnormality found in the PARK15 patients from the Dutch and Italian families is the depletion of the FBXO7 isoform 1 (NM_012179), which normally is located in the cell nucleus [15]. Forty-eight hours after transfection of EGFP tagged FBXO7 isoform 1 constructs, cells were analyzed by fluorescent microscopy. Although Cys52 FBXO7 protein displayed nuclear and cytosolic staining pattern similar to Tyr52, a significantly stronger green (FBXO7 fusion protein) relative to blue (nuclei staining) fluorescence signal was observed in Cys52 FBXO7 cells (1.60 vs. 2.57, *p* = 0.015; Fig. 2A). To further examine the transiently expressed FBXO7-EGFP fusion protein, protein blotted with FBXO7 antibody was performed. As shown in Fig. 2B, FBXO7-EGFP fusion protein in the expected size range for Tyr52 and Cys52 constructs was observed. However, the protein expression level of Cys52 FBXO7 was increased compared with the Tyr52 FBXO7 (211%, *p* = 0.016). The stability of Cys52 variant was further examined by a cycloheximide (200 µg/ml) chase experiment. While the Tyr52 protein was degraded to 68%, 18%, 11%, 9% and 7% left after 6, 12, 24, 36, and 48 hr of protein synthesis blocking, reduced rates of decay were observed for Cys52 variant (90%, 78%, 72%, 52%, and 21% remained, respectively) (Fig. 2C).

### Homology modeling of Cys52 FBXO7

**Figure 3:**
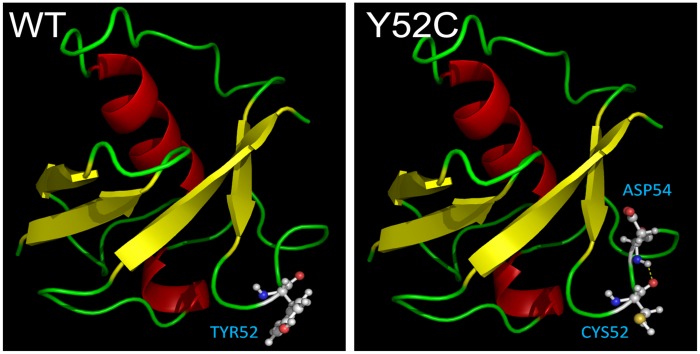
The homology models of Tyr52 (WT) FBXO7 and Cys52 (Y52C). Protein models were shown in secondary representation. Tyr52 and Cys52 residue were shown in amino acid structures. The hydrogen bond interaction was presented in yellow dash line.

To understand the structure-based information of Cys52 variant in FBXO7, homology modeling of Tyr52 and Cys52 FBXO7 was performed. After energy minimization, the modeled structures for Tyr52 (WT) and Cys52 (Y52C) were shown in Fig. 3. The potential energy of Tyr52 and Cys52 mutant was −2463.854 and −2471.736 kcal/mol, indicating Cys52 FBXO7 exhibited a more stable feature than Tyr52 FBXO7. According to hydrogen-bond (H-bond) computing analysis, the H-bond interaction of Tyr and Cys52 was shown. In the Tyr52 model, Tyr52 did not form any H-bonds with adjacent residue. On the other hand, H-bond formed by the Cys52 with the Asp54 causes decrease of the local energy.

### FBXO7 regulating TRAF2 abundance through ubiquitinating TRAF2

**Figure 4:**
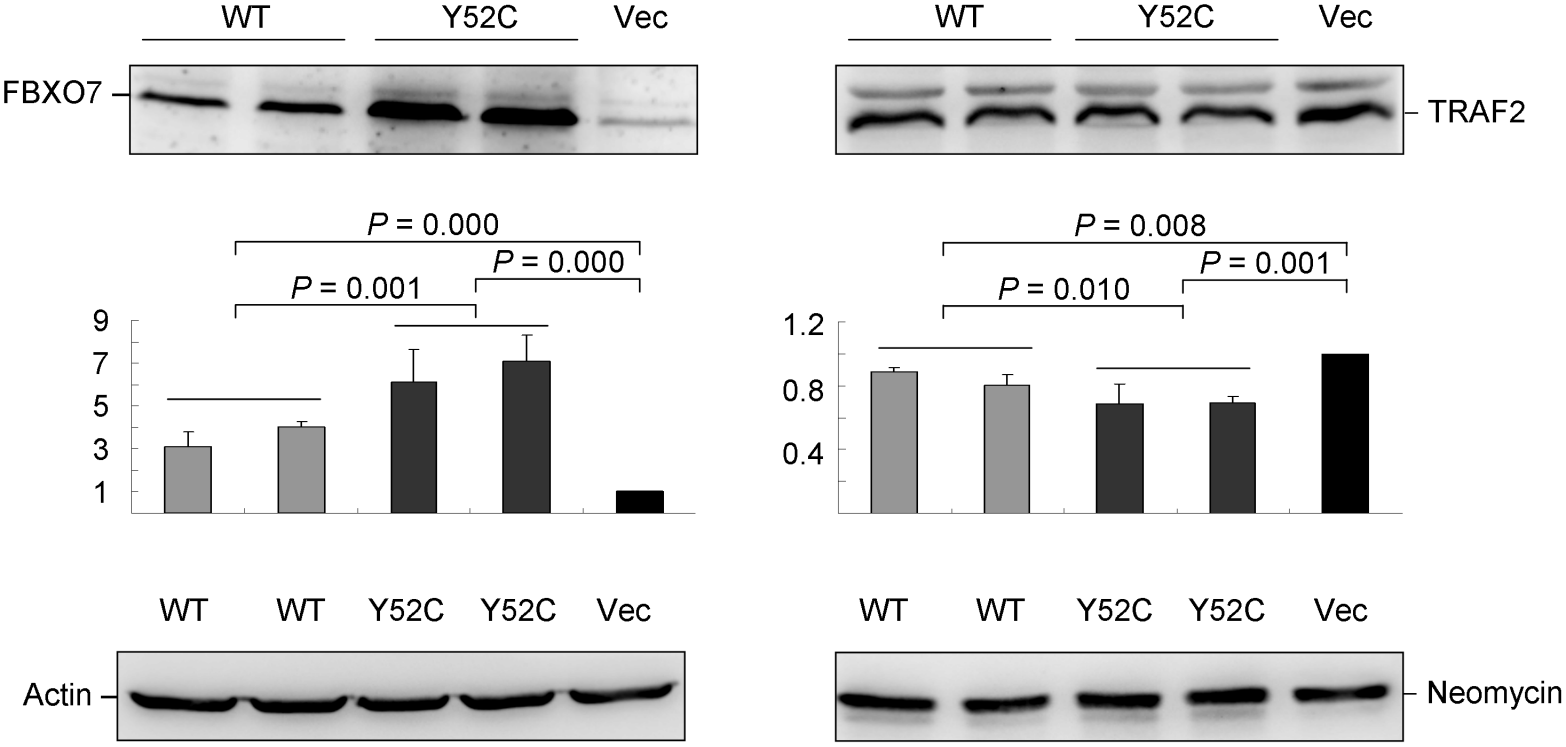
Western blot analysis of NF-κB signaling pathway protein TRAF2. HEK-293T cells were tranfected with Tyr52 or Cys52 FBXO7 construct and harvested after two days. Transfected FBXO7-EGFP or endogenous TRAF2 protein was detected using specific antibodies and anti-actin and anti-neomycin antibodies as the loading and the transfection control, respectively. Data are represented as the means ± S.D. of three separate experiments.

Through binding and mediating ubiquitin conjugation to TRAF2 and cIAP1, FBXO7 was identified as a negative regulator of NF-κB signaling [12]. To assess Cys52's effect on TRAF2 abundance, Tyr52 or Cys52 FBXO7 cDNA plasmid was transfected into HEK-293T cells and protein was blotted with TRAF2 and FBXO7 antibodies (Fig. 4). Compared with the endogenous FBXO7 level, Tyr52 or Cys52 cDNA transfection significantly increased FBXO7 abundance (3.11–7.11 folds, *p*<0.001). The FBXO7 level of Cys52 was significantly higher than that of Tyr52 (6.15–7.11 vs. 3.11–4.01, *p* = 0.001). Accompanying that, TRAF2 protein was significantly decreased in Tyr52 or Cys52 FBXO7 cells (0.69–0. 0.89, *p* = 0.008–0.001). In Cys52 cells, the TRAF2 expression level was significantly lower than that of Tyr52 cells (0.69–0.70 vs. 0.80–0.89, *p* = 0.010).

**Figure 5:**
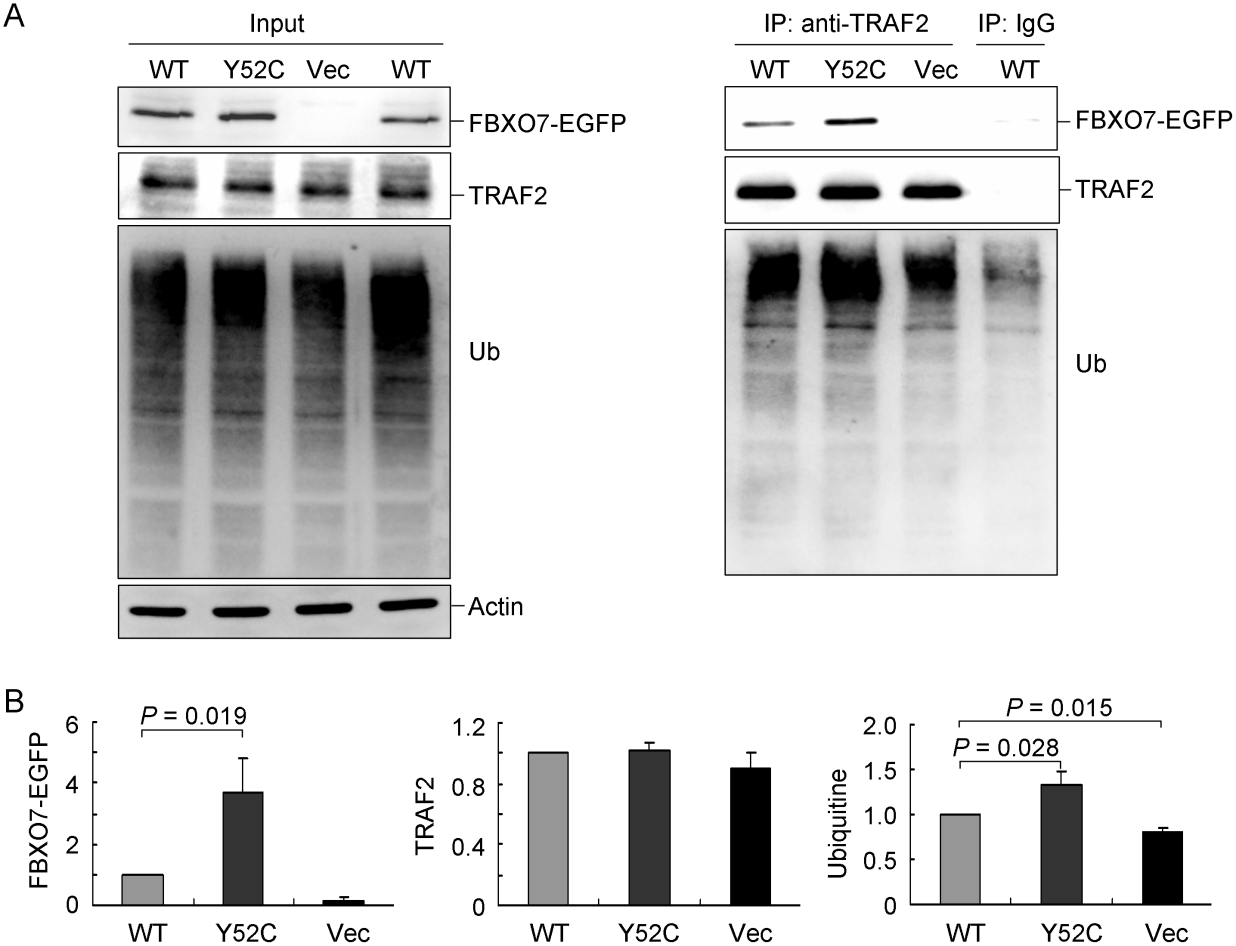
Co-immunoprecipitation of Tyr52 or Cys52 FBXO7-EGFP protein and ubiquitination of TRAF2. (A) HEK-293T cells were transiently transfected with Tyr52 or Cys52 FBXO7-EGFP construct. After 48 h, cell lysates were prepared (Input, left panel) and immunoprecipitations (IP, right panel) were performed with anti-TRAF2 antibody. Normal IgG was used as a negative control for IP. Cell lysates and immunoprecipitates were analyzed with anti-EGFP, anti-TRAF2, anti-ubiquitin or anti-actin antibody. (B) Quantification of immunoprecipitated FBXO7-EGFP, TRAF2 and ubiquitin in HEK-293T cells transiently transfected with Tyr52 or Cys52 FBXO7-EGFP construct for 2 days. Data are represented as the means ± S.D. of three separate experiments.

Furthermore, to examine whether Cys52 exerts effects on TRAF2 ubiquitination, proteasome inhibitor MG-132 (5 µM) was applied to Tyr52 or Cys52 FBXO7-EGFP expressing HEK-293T cells and co-immunoprecipitation was performed (Fig. 5A). The results showed a significant increase in FBXO7-TRAF2 interaction (367% vs. 100%, *p* = 0.019, Fig. 5B) in Cys52-expressing cells as compared to Tyr52-expressing cells. Moreover, a significant increase in TRAF2 ubiquitination was observed in Cys52 cells as compared to Tyr52 cells (132% vs. 100%, *p* = 0.028) and in Tyr52 cells as compared to vector-transfected cells (100% vs. 80%, *p* = 0.015) (Fig. 5B). These results suggest that FBXO7 may decrease TRAF2 abundance through promoting TRAF2 ubiquitination.

### Suppression or over-expression of FBXO7 affecting cell survival upon MPP^+^ treatment

**Figure 6:**
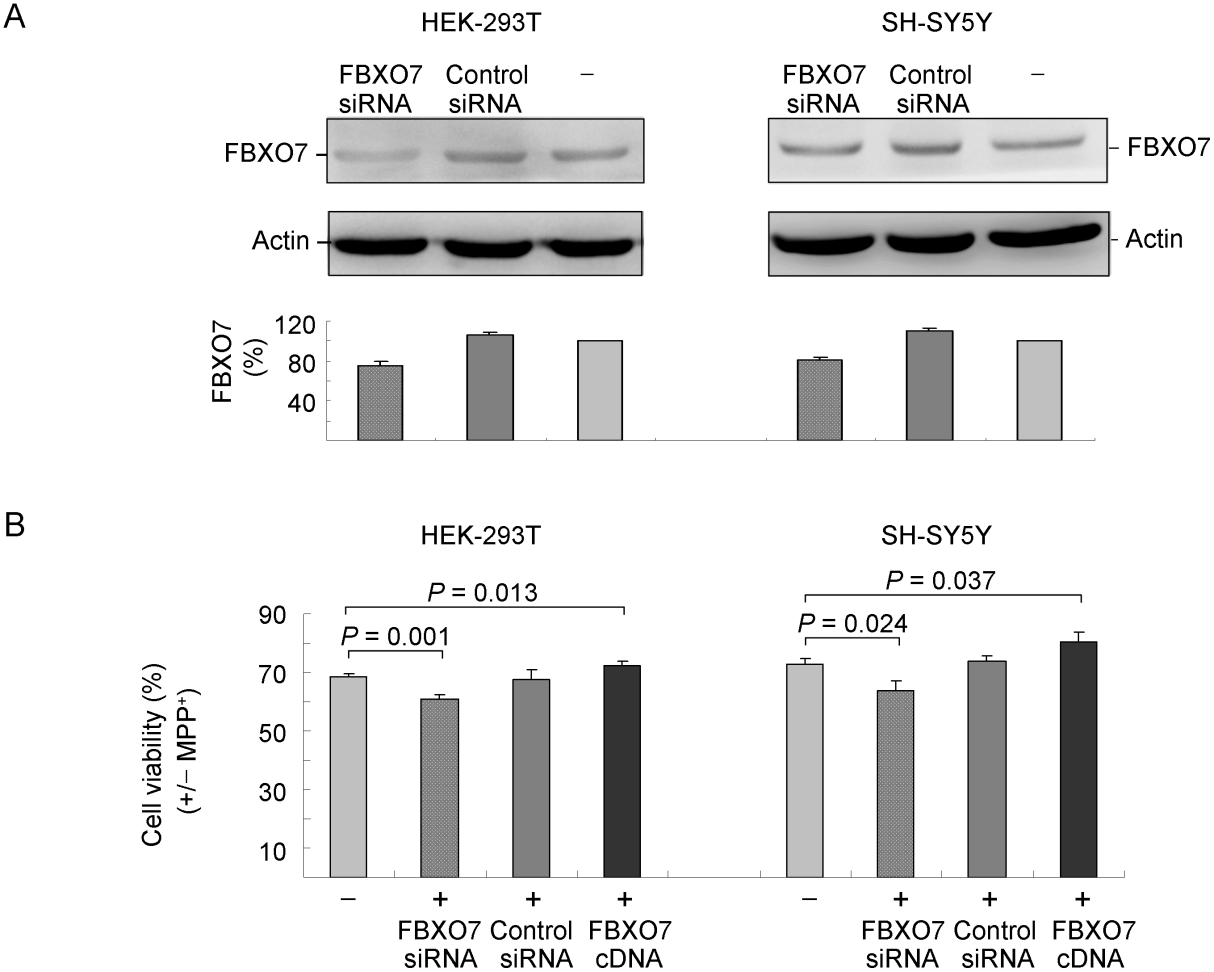
Toxic effects of MPP^+^ in FBXO7 suppressed or over-expressed cells. (A) HEK-293T and SH-SY5Y cells were transfected with FBXO7-specific or control siRNA. After 48 h, cell lysates were prepared and Western blot analysis was performed using FBXO7 and anti-actin (as loading control) antibodies. (B) HEK-293T and SH-SY5Y cells were transfected with siRNA (FBXO7-specific or control), FBXO7 cDNA or not (−). After 24 hr, cells were treated with MPP^+^ (300 µM for HEK-293T or 2 mM for SH-SY5Y) and toxic effects of MPP^+^ were monitored at 24 hr by MTT assay.

To gain further insight into the possible role of FBXO7 protein involved in PD, the mitochondrial inhibitor MPP^+^ was applied to FBXO7 knockdown or over-expressing cells for 24 hours. MTT assay was performed to evaluate the cell viability. As shown in Fig. 6A, the knock down of FBXO7 was confirmed as 76% (*p* = 0.005–<0.001) and 81% (*p* = 0.004–0.001) of FBXO7 were seen in FBXO7 siRNA-transfected HEK-293T and SH-SY5Y cells, respectively, as compared to control siRNA-transfected (106%∼110%) or untransfected (100%) cells. Fig. 6B examined if suppression or over-expression of FBXO7 affects cell survival upon MPP^+^ treatment. With 300 µM MPP^+^ treatment, the cell viability of HEK-293T cells expressing FBXO7 siRNA was 61% of the untreated cells, which is significantly lower than that of untransfected (69%, *p* = 0.001) or control siRNA transfected (68%, *p* = 0.033) HEK-293T cells. A similar result was obtained in SH-SY5Y cells treated with 2 mM MPP^+^, as suppression of FBXO7 expression also significantly decreased the cell viability (64%) as compared to untransfected (73%, *p* = 0.024) or control siRNA transfected (74%, *p* = 0.018) cells. Conversely, significantly increased cell viability was observed with MPP^+^ treatment in FBXO7-EGFP over-expressing cells compared to untransfected cells (HEK-293T: 73% vs. 69%, *p* = 0.013; SH-SY5Y: 81% vs. 73%, *p* = 0.037).

### SH-SY5Y cell model

**Figure 7:**
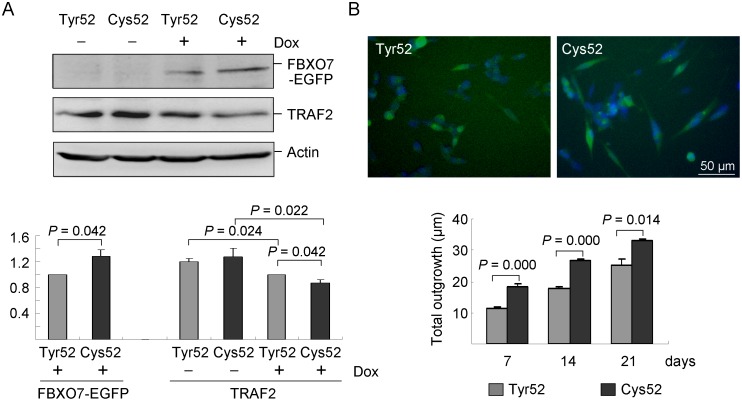
Flp-In SH-SY5Y cells with induced FBXO7 expression and neuronal phenotype. (A) Western blot analysis of FBXO7-EGFP protein level using FBXO7 antibody and anti-actin antibody as loading control after 2 days induction with doxycycline (+ Dox) or not (- Dox). (B) Representative microscopic images of neuronal differentiated Tyr52 and Cys52 cells (for 21 days). Nuclei were counterstained with DAPI (blue). Neuronal total outgrowth was quantified in Tyr52 and Cys52 cells with induced differentiation for 7–21 days. Data are represented as the means ± S.D. of three separate experiments.

To test the effect of Cys52 on neuronal phenotype, we constructed Flp-In SH-SY5Y cells with Tyr52 or Cys52 FBXO7-EGFP expression in an inducible fashion. Immunoblot analysis showed that the FBXO7 protein level was significantly increased in Cys52 cells as compared to that of Tyr52 cells after induction with doxycycline (+ Dox) for 2 days (128%, *p* = 0.042) (Fig. 7A). Compared to the non-induced cells (- Dox), TRAF2 protein level was significantly decreased in both Tyr52 (100% vs. 120%, *p* = 0.024) and Cys52 (87% vs. 128%, *p* = 0.022) FBXO7-EGFP expressed cells. The difference in TRAF2 abundance between Tyr52 and Cys52 FBXO7-EGFP expressed cells was also significant (100% vs. 87%, *p* = 0.042). These FBXO7 cells were induced for differentiation with retinoic acid [16] for 7 to 21 days. Representative fluorescence microscopy images of cells differentiated for 21 days are shown in Fig. 7B. Significantly more total outgrowth in Cys52 cells was observed compared to Tyr52 cells after differentiation for 7–21 days (131–165%, *p* = 0.014–<0.001).

## Discussion

Up to now, only four different types of *FBXO7* mutations (T22M, R378G, R498X, IVS7+1G/T) have been reported to be responsible for parkinsonian-pyramidal disease, which has been designated as the cause of PARK15 [4], [5], [6]. Recently, two missense substitutions (p.Ile87Thr and p.Asp328Arg) were found in two EOPD patients in Taiwan [7]. We did not detect any mutation in our EOPD patients, which is compatible with the previous results of studies showing rare pathogenic mutations of the *FBXO7* gene in typical PD patients of Chinese ethnicity [1], [7]. Nevertheless, our method using cDNA sequencing may miss some mutations in the non-coding and regulatory elements of the *FBXO7* gene. Also variants which result in a truncated protein or nonsense mediated decay would not be identified.

Although the role of FBXO7 in neurons is still not known, because it is a part of SCF ubiquitin ligase complex, its function in the ubiquitin-mediated protein degradation is implicated [9]. Impaired ubiquitin-mediated protein degradation has been found in sporadic PD [17], autosomal dominant PARK1 [18], and autosomal recessive PARK2 [19]. Therefore, it is postulated that *FBXO7* mutations may compromise the ubiquitin-proteasome function and cause neuronal dysfunction in PD. In this study, we showed that the Y52C AG genotype or G allele of *FBXO7* conferred a reduced susceptibility to Chinese PD when the data from our study and Luo's [1] were combined. However, as Y52C G allele is rare and the difference in frequency is small (0.4% in PD and 1.2% in controls), the genetic evidence is very limited and the findings may be due to chance alone. While *FBXO7* mutations typically causes autosomal recessive parkinsonism with pyramidal tract signs, the Y52C AG genotype or G allele in this study appears to provide a protective effect in a dominant mode. T22M, R378G, R498X mutations resulted in decreased stability of FBXO7 protein and T22M caused loss of its nuclear activity, both of which may jeopardize the neuronal function [15]. Using the program SWISS-MODEL, we showed that the Cys52 formed H-bond with the Asp54 causing decreased local energy, which may increase the stability of the protein. The increased stability of FBXO7 protein consequent to Cys52 was further confirmed by cycloheximide chase experiment (Fig. 2). These results suggest that Cys52 may play a protective role in PD via increasing stability of FBXO7 protein, which is in contrast to the decreased stability and loss of function caused by T22M, R378G, and R498X [15]. Indeed, knock down of *FBXO7* leading to dopaminergic neuronal dysfunction and cell loss and locomotor deficits that are improved by apomorphine, have been shown in zebrafish [20]. The study in zebrafish further suggests that *FBXO7* plays an important role in the development of dopaminergic neurons and its loss of function caused by mutations may be responsible for the phenotype of PD.

FBXO7 can interact with three proteins including hepatoma upregulated protein, cIAP1, and the proteasome inhibitor protein PI31 [11], [21], [22]. FBXO7 was also reported to enhance activity of cyclin D/cdk6 that plays an important role in regulating neuronal death processes [23]. How these interactions may contribute to the pathogenesis of PD remains to be clarified.

More emerging evidence has suggested that neuroinflammation is involved in the pathogenesis of PD through inflammatory mediators such as TNFα, nitric oxide (NO), IL-6, and IL-1β [13]. NF-κB activation is required for all of these inflammatory mediators to be produced by microglial cells [14]. Recently, few NF-κB inhibitors have been applied to the therapeutic approaches of several chronic inflammatory diseases including PD [13]. Interestingly, FBXO7 protein has been shown to be a negative regulator of NF-κB signaling pathway, through binding to and ubiquitinating TRAF2 and cIAP1, which would lead to decreased RIP1 ubiquitination and NF-κB activity [12]. To further investigate the role of FBXO7 in NF-κB signaling, Cys52 variant was expressed in HEK-293T and SH-SY5Y cells and TRAF2 (NF-κB signaling protein) was examined in this study. As shown in Fig. 4–6, structurally stable Cys52 FBXO7 facilitated the degradation of TRAF2 protein via increasing TRAF2 ubiquination, which may theoretically further lower the NF-κB activity. Although the NF-κB activity was not evaluated in this study, the results provide some evidence that Cys52 variant may play a protective role in PD pathogenesis. Whether other NF-κB signaling proteins such as RIP1 are also regulated by FBXO7 needs to be investigated in future studies.

In conclusion, gene mutations may be rare in Chinese early-onset Parkinsonism patients. We have shown that Y52C polymorphism of FBXO7 may contribute to reduced PD susceptibility in Chinese population. However, additional case–control studies are needed to establish whether FBXO7 variants truly play a role in PD.

## Materials and Methods

### Ethics statement

This study was performed according to a protocol approved by the Institutional Review Board of Chang Gung Memorial Hospital, and all examinations were performed after obtaining written informed consents.

### Subjects

A total of 516 unrelated Taiwanese PD subjects (45.0% females) were recruited from the neurology clinics of Chang Gung Memorial Hospital (CGMH). All patients were diagnosed by two neurologists specialized in movement disorders (Y.-R. Wu and C.-M. Chen) with probable idiopathic PD according to the published criteria [24], which includes substantial and sustained response to levodopa or a dopamine agonist. Subjects with prior history of multiple cerebrovascular events or other causes of parkinsonian symptoms (e.g. brain injury or tumor, encephalitis, antipsychotic medication) were excluded. The mean age at onset (AAO) of PD was 62.0±11.5 years, ranging between 19 and 93 years. For juvenile PD patients (AAO≤50), mutations in the *Parkin*, *PINK1*, *DJ-1, ATP13A2* and G2019S *LRRK2* were excluded ([25], [26], [27] and unpublished results). A group of 516 normal controls without neurodegenerative diseases were recruited from the same ethnic community. Control subjects (50.2% females) had mean age at examination of 60.9±12.3 years, ranging between 20 and 92 years. All examinations were performed after obtaining written informed consent from patients and control individuals. This study was approved by the Institutional Review Board of CGMH.

### Genetic analysis

Genomic DNA was extracted from peripheral blood leucocytes using the standard protocols. For PD patients with onset ≤50 (n = 80, mean age at onset 43.7±0.7 years, 33.7% females), RNA was extracted using PAXgene Blood RNA Kit (PreAnalytiX). The RNA was DNase (Stratagene) treated, quantified, and reverse-transcribed to cDNA using High Capacity cDNA Reverse Transcription Kit (Applied Biosystems). Using polymerase chain reaction (PCR) with designed primers and conditions (Table 1), the 1955-bp amplified *FBXO7* cDNA was gel purified and sequenced directly using the ABI PRISM 3130 Genetic Analyzer (Applied Biosystems). The identified Y52C and M115I variants were verified by genomic DNA PCR and sequencing. For population screening, the Y52C and M115I were examined using the *Pst*I and *Pae*I (gain of sites) restriction enzymes, respectively (Table 1). The digested PCR products were visualized with ethidium bromide after electrophoresis in 2.2% or 1.6% agarose gel.

### FBXO7 cDNA constructs

Using the designed primers to remove translation termination codon (Table 1), the full-length *FBXO7* cDNA fragments from an individual heterozygous for Cys52 were cloned into pGEM-T Easy vector (Promega) and sequenced. The 1.7 kb *Hin*dIII (added in the forward primer)–*Age*I (added in the reverse primer) fragments were removed from pGEM-T Easy vector and ligated into the corresponding sites of pEGFP-N1 (Clontech) to generate Tyr52 and Cys52 *FBXO7* cDNA in-frame fused to the EGFP gene. The resulting EGFP-tagged FBXO7 constructs were used in transient expression studies for confocal microscopy examination, FBXO7 stability and anti-TRAF2 co-immunoprecipitation. Additionally, the *Hin*dIII-*Xho*I fragments containing FBXO7 were ligated into pcDNA3.1/V5-His (Invitrogen) to generate Tyr52 and Cys52 *FBXO7* cDNA in-frame fused to the V5-His for Western blot analysis of NF-κB signaling pathway protein TRAF2.

### Cell cultivation and transfection

Human embryonic kidney (HEK)-293T (ATCC No. CRL-11268) cells were cultivated in Dulbecco's modified Eagle's medium containing 10% fetal bovine serum (FBS) in a 37°C humidified incubator with a 5% CO_2_ atmosphere. Cells were plated into 6-well (6×10^5^/well) dishes, grown for 20 hr and transfected by the lipofection method (GibcoBRL) with EGFP-tagged FBXO7 constructs (4 µg/well). The cells were grown for 48 hr for the protein studies. To evaluate the stability of FBXO7 protein, protein synthesis inhibitor cycloheximide (200 µg/ml) was added 24 hr after transfection for 0, 6, 12, 24, 36, and 48 hr before protein preparation. For immunoprecipitation studies, proteasome inhibitor MG-132 (5 µM) was added 24 hr after transfection for 24 hr before protein preparation.

### Confocal microscopy examination

For visualizing intracellular FBXO7-EGFP protein, transfected cells on coverslips were stained with 4′-6-diamidino-2-phenylindole (DAPI) to detect nuclei. The stained cells were examined for dual fluorescent imaging using a Leica TCS confocal laser scanning microscope.

### siRNA transfection and cell viability assay

Human neuroblastoma SH-SY5Y cells (ATCC No. CRL-2266) were maintained in DMEM F12 supplemented with 10% FBS at 37°C in an atmosphere containing 5% CO_2_. For FBXO7 knockdown, siRNA specifically targeting FBXO7 (J-013606-06, Thermo Scientific) was transfected into in HEK-293T (by lipofection) or SH-SY5Y cells (by 4D-Nucleofector System, Lonza). For transfection of SH-SY5Y, cells (2.5×10^5^/48-well) were centrifuged at 90×g for 10 min at room temperature. The cell pellets were resuspended in 4D-Nucleofector Solution (100 µL) with siRNA (100 nM) or FBXO7-EGFP (250 ng) and CA-137 appropriate program was selected. The transfected cells were plated in 48-well plate and grown for 48 hr for protein analysis. Alternatively, after transfection for one day, cells were treated with freshly prepared mitochondrial inhibitor MPP^+^ (1-methyl-4-phenylpyridinium ion, 300 µM for HEK-293T or 2 mM for SH-SY5Y cells). After one day, 20 µL MTT [3-(4,5-dimethylthiazol-2-yl)-2,5-diphenyltetrazolium bromide, 5 mg/mL in PBS, Sigma] was added to cells and incubated for 2 hr. The absorbance of the insoluble purple formazan product was measured at 570 nm by a Bio-Tek μQuant Universal Microplate Spectrophotometer.

### Western blot analysis

Cells were lysed in hypotonic buffer (20 mM HEPES pH 7.4, 1 mM MgCl_2_, 10 mM KCl, 1 mM DTT, 1 mM EDTA pH 8.0) containing the protease inhibitor mixture (Sigma). After sonication and sitting on ice for 20 min, the lysates were centrifuged at 14,000×g for 30 min at 4°C. Protein concentrations were determined using the Bio-Rad protein assay kit, with albumin as standards. Total proteins (25 µg) were electrophoresed on 10% SDS-polyacrylamide gel and transferred onto nitrocellulose membrane (Schleicher and Schuell) by reverse electrophoresis. After being blocked, the membrane was stained with anti-FBXO7 (1∶3000 dilution, Abnova), anti-TRAF2 (1∶500 dilution, Santa Cruz), anti-neomycin (1∶1000 dilution, Millipore), anti-tubulin (1∶10000 dilution, GeneTex), anti-GAPDH (1∶1000 dilution, MDBio), anti-actin (1∶10000 dilution, Millipore), anti-EGFP (1∶200 dilution, Santa Cruz), or anti-ubiquitin (1∶1000 dilution, Dako) antibody. The immune complexes were detected using horseradish peroxidase-conjugated goat anti-mouse (Jackson ImmunoResearch) or goat anti-rabbit (Rochland) IgG antibody (1∶10000 dilution) and Immobilon Western Chemiluminescent HRP substrate (Millipore).

### Immunoprecipitation

Total protein from MG-132-treated FBXO7-EGFP-transfected cells was prepared using IP lysis buffer (25 mM Tris-HCl pH 7.4, 150 mM NaCl, 1% NP-40, 1 mM EDTA, 5% glycerol) containing the protease inhibitor mixture. After quantification, proteins (100 µg) were immunoprecipitated with mouse TRAF2 primary antibody (2 µg, Santa Cruz) conjugated to protein G beads (Millipore). The beads-proteins-antibody mixtures were washed three times with IP lysis buffer and the immunoprecipitated proteins were eluted by 1× SDS sample loading buffer (50 mM Tris pH 6.8, 2% SDS, 10% glycerol, 2.5% β-mercaptoethanol, 0.005% bromophenolblue), separated on 10% SDS-polyacrylamide gel, transferred onto nitrocellulose membrane and probed with indicated antibodies as described.

### FBXO7 SH-SY5Y cell lines generation

The SH-SY5Y-derived FIp-In host cells [28] and Flp-In T-REx System (Invitrogen) was used to generate stably induced SH-SY5Y cell lines exhibiting tetracycline-inducible expression of Tyr52 and Cys52 FBXO7. Briefly, the SH-SY5Y host cells were co-transfected with pOG44 plasmid (constitutively expressed the Flp recombinase) and pcDNA5/FRT/TO-FBXO7-EGFP plasmid according to the supplier's instructions. These cell lines were grown in medium containing 5 µg/ml blasticidin and 100 µg/ml hygromycin. Doxycycline (dox, 5 µg/ml) was added to induce EGFP-tagged FBXO7 expression for two days. The proteins were prepared for Western blotting using antibody to FBXO7 or actin as described. Neuronal phenotypes were examined after induced differentiation with retinoid acid (10 µM) and induced expression of FBXO7 for 7 to 21 days. The morphologic differentiation of Tyr52 and Cys52 SH-SY5Y cells including total outgrowth, processes, and branches was assessed by using Metamorph microscopy automation and image analysis software (Molecular Devices).

### Statistical analysis

The genotype frequency data and the expected genotypic frequency under random mating were computed and Chi-square tested for Hardy-Weinberg equilibrium using standardized formula. The genotype and allele association analysis was carried out using the Chi-square test. Odds ratios with 95% confidence intervals (95% CI) were calculated to test association between genotype/allele and disease. Given the observed Y52C G allele frequency of 0.0078 (0.0095, in combined data from Taiwan and China) and a total of 1032 (1367, combined subjects from Taiwan and China) subjects in the present study, at significance level of 0.05, we had power greater than 0.8 to identify an association when the allele genetic effect size was greater than 3.1 (2.5). Given the observed M115I G allele frequency of 0.286 and 1032 subjects in the present study, at significance level of 0.05, we had power greater than 0.8 to identify an association when the allele genetic effect was greater than 1.5.

For statistical analysis of microscopy images, immunoblots and cell viability assays, data were expressed as the means ± standard deviation (SD). Three independent experiments were performed and non-categorical variables were compared using the Student's *t*-test. All *p*-values were two-tailed, with values of *p*<0.05 being considered significant.

### Homology modeling

We modeled the three dimensional structures of the Tyr52 and Cys52 FBXO7 proteins by comparative methods and energy minimization using the program SWISS-MODEL [29]. The 2.9-Å coordinate set for the crystal structure of human UBC protein (PDB code 2ZVO, chain A) served as the template for modeling the residue 1–79 of human FBXO7. The energy computation was done with the GROMOS96 [30] implementation of Swiss-PdbViewer. The resulting FBXO7 three-dimensional models were manipulated and rendered in PyMOL (The PyMOL Molecular Graphics System, Version 1.2r3pre, Schrödinger, LLC).
